# Targeted Sterically Stabilized Phospholipid siRNA Nanomedicine for Hepatic and Renal Fibrosis

**DOI:** 10.3390/nano6010008

**Published:** 2016-01-05

**Authors:** Fatima Khaja, Dulari Jayawardena, Antonina Kuzmis, Hayat Önyüksel

**Affiliations:** Department of Biopharmaceutical Sciences (M/C 865), College of Pharmacy, University of Illinois at Chicago, 833 South Wood St., Chicago, IL 60612-7231, USA; fatima.a.khaja@gmail.com (F.K.); djayaw2@uic.edu (D.J.); tkuzmis@uic.edu (A.K.)

**Keywords:** siRNA, sterically stabilized phospholipid nanoparticles, galactosamine, hepatic stellate cells, fibrosis

## Abstract

Since its discovery, small interfering RNA (siRNA) has been considered a potent tool for modulating gene expression. It has the ability to specifically target proteins via selective degradation of messenger RNA (mRNA) not easily accessed by conventional drugs. Hence, RNA interference (RNAi) therapeutics have great potential in the treatment of many diseases caused by faulty protein expression such as fibrosis and cancer. However, for clinical application siRNA faces a number of obstacles, such as poor *in vivo* stability, and off-target effects. Here we developed a unique targeted nanomedicine to tackle current siRNA delivery issues by formulating a biocompatible, biodegradable and relatively inexpensive nanocarrier of sterically stabilized phospholipid nanoparticles (SSLNPs). This nanocarrier is capable of incorporating siRNA in its core through self-association with a novel cationic lipid composed of naturally occuring phospholipids and amino acids. This overall assembly protects and delivers sufficient amounts of siRNA to knockdown over-expressed protein in target cells. The siRNA used in this study, targets connective tissue growth factor (CTGF), an important regulator of fibrosis in both hepatic and renal cells. Furthermore, asialoglycoprotein receptors are targeted by attaching the galactosamine ligand to the nanocarries which enhances the uptake of nanoparticles by hepatocytes and renal tubular epithelial cells, the major producers of CTGF in fibrosis. On animals this innovative nanoconstruct, small interfering RNA in sterically stabilized phospholipid nanoparticles (siRNA-SSLNP), showed favorable pharmacokinetic properties and accumulated mostly in hepatic and renal tissues making siRNA-SSLNP a suitable system for targeting liver and kidney fibrotic diseases.

## 1. Introduction

Small interfering RNA (siRNA) is a potent tool for modulating gene expression owing to its high specificity to target mRNA, not easily accessed by traditional drug molecules [1]. Hence, RNAi therapeutics have demonstrated potential as a more personalized approach in the treatment of many life threatening diseases [2] caused by faulty protein expression, such as malignancies, fibrosis and amyloidosis [3,4,5]. However, as a naked molecule, siRNA is susceptible to degradation, rapid clearance and a wide bio-distribution due to its small size and high negative charge [6,7,8]. On the other hand, developing carrier systems that can protect and target siRNA to its intended site of action have demonstrated manufacturing and safety challenges [9,10].

Whether RNAi therapeutics will make it from bench to bedside will largely depend on the improvement of siRNA molecule’s targetability and pharmacokinetics in terms of plasma stability and circulation time, as well as specific cellular uptake [11]. In general, systemically administered naked siRNA molecule faces extracellular and intracellular barriers [12]. Free siRNA molecules are exposed to serum nucleases and phagocyte uptake which markedly reduce their biological half-life. Moreover, once introduced locally the negative charge of the plasma membrane as well as the extracellular matrix (ECM) hinders these molecules from reaching their target and exerting action [13].

Intracellular barriers are also crucial determinants of the efficiency of a carrier encapsulating siRNA molecules as these are engulfed by endocytosis. The carriers face the challenge of disassembling in a timely manner and escaping the endosome in order to deliver their siRNA cargo to target mRNA, located in the cytoplasm [9].

Another challenge of siRNA therapeutics is off-target effects or suppression of normal genes after non-targeted systemic administration which can lead to damaging or undesired cell transformation [14]. Recent reports have also demonstrated that free siRNA can initiate interferon responses via toll-like receptor 7 (TLR-7) leading to cell death in culture [1]. In addition, phagocytic cells present in bloodstream and extravascular tissues can detect and interact with foreign siRNA molecules causing the activation of further immune responses [9].

Over the last decade, researchers have been investigating siRNA modification approaches and carrier system development that overcomes its delivery barriers. An ideal vehicle should have the capability to completely encapsulate and protect the siRNA cargo against enzymatic degradation and have an appropriate size to allow extravasation and retention at the target site, while preventing renal clearance. It should also possess appropriate surface properties that prevent serum protein interaction and allow efficient uptake by target cells while evading phagolysosomes. Other desired characteristics that facilitate clinical application are easy functionalization with targeting ligands to enhance tissue specificity, biocompatibility and reduce toxicity [9,15].

Although much progress have been accomplished using viral vectors, modified siRNA, and various nanocarriers, their usage as a clinically applicable delivery system is still arguable due to multiple drawbacks related to safety and stability concerns [16]. On the other hand, synthetic cationic materials have demonstrated some potential as non-viral siRNA delivery vehicles [15]. Cationic polymers offer several benefits including the ability to facilitate complex formation with negatively charged siRNA molecules through electrostatic interactions, cellular uptake, and proton sponge-mediated endosomal escape [17]. However, disadvantages of cationic carriers include high toxicity due to cell membrane integrity alteration and high immunogenicity [18]*.*

Lipid nanoparticles in general and phospholipids specifically have been recognized as one of the most promising delivery systems for siRNA due to their biocompatibility, relative ease of large scale production and the recent approvals to be used in clinical trials [19,20]. Phospholipids are amphiphilic molecules that form spontaneous bilayer structures upon dispersion in water [21] entrapping the dispersed hydrophilic payload within the aqueous core of the formed structure. Therefore, chemical modification of the head groups of these molecules with cationic arginine molecules should promote entrapment of negatively charged siRNA through electrostatic interactions, making them promising components of siRNA nanocarriers [22,23].

Connective tissue growth factor (CTGF) is considered the master switch in chronic fibrotic diseases [24,25] and provides a unique strategy for siRNA targeted therapeutics [26]. Following chronic organ injury, CTGF is over expressed as a part of the wound healing response exerting its own pro-fibrotic effect as well as facilitating production of profibrotic cytokine transforming growth factor β1 (TGF-β1). These two factors work synergistically to activate endothelial cells to exert a phenotype of proliferative myofibroblasts, in turn, causing accumulation of collagen and other proteins in the surrounding ECM, thus affecting the organ morphology and function [25,27]. Downregulation of CTGF expression has shown to be an effective strategy for the reversal of endothelial cell activation and accumulation of fibrotic ECM [28,29]. Recently it has been demonstrated that targeting CTGF by an siRNA based cationic solid lipid nanoparticle in liver fibrosis successfully reversed symptoms of fibrosis as well as reduced content of key downstream mediators regulating this disease [30]. Furthermore, CTGF has been targeted via another delivery vehicle for reducing cardiac fibrosis indicating the important role of the protein during the pathogenesis of disease [31].

In this study, we formulated a biocompatible and relatively inexpensive sterically stabilized phospholipid nanoparticles (SSLNPs) composed of naturally occuring phospholipids and amino acid components in addition to the US Food and Drug Administration (FDA) approved DSPE-PEG_2000_ monomers [32]. This nanocarrier is designed to effectively load and deliver sufficient amounts of siRNA against CTGF to hepatocytes or renal tubular epithelial cells through passive and active targeting mechanisms established by the nanosize of the particle and surface conjugation with galactosamine (GalN). GalN is known to target asialoglycoprotein receptors, expressed on the surface of hepatocytes [33,34]. Scientific evidence suggests that receptor mediated endocytosis results in the internalization of the siRNA and sequence-specific degradation of CTGF mRNA causing the down-modulation of CTGF activity. This effect, in turn shifts the TGF-β1/Bone morphogenic protein 7 (BMP-7-a natural antagonist of TGF-β1) balance in the direction of anti-fibrosis, *i.e.*, inhibiting ECM synthesis and increasing its degradation [25].

In the present study, we first performed physicochemical characterization and stability studies of siRNA encapsulated in SSLNPs. Then, we demonstrated the low cytotoxicity of SSLNP on different cell lines as well as their significant uptake in cell culture. Efficacy of developped siRNA-SSLNP nanomedicine against CTGF and the reversal of endothelial cell activation was demonstrated through the downregulation of key protein players of fibrosis *in vitro*.

Finally, bio-distribution (BD) and pharmacokinetic (PK) studies in mice were performed to confirm the potential of using these nanocarriers for targeted delivery to liver and kidney tissues. To achieve steric stability and improve BD/PK properties [35] of our carrier, we used a polyethylene glycol conjugated (PEGylated) lipid, which is approved for human use by FDA in another pharmaceutical product. We believe that the entire particle surface is covered by PEG so that possible immune reaction and activation of the complement system [36,37] is minimal.

## 2. Results and Discussion

### 2.1. Results

#### 2.1.1. Optimization of SSLNPs for siRNA Delivery

**Figure 1:**
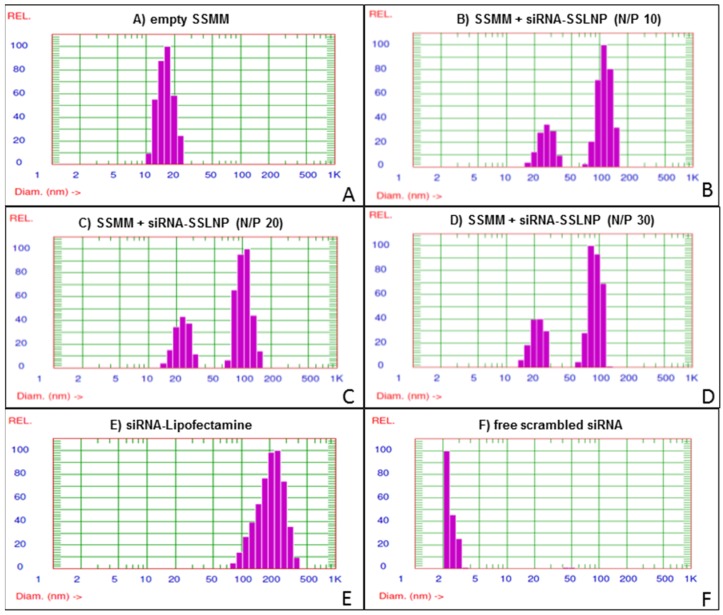
small interfering RNA in sterically stabilized phospholipid nanoparticles (siRNA-SSLNP) optimization *in vitro*: Representative particle size distribution of (**A**) Unimodal size distribution of empty SSMM (sterically stabilized mixed micelles); (**B**) Empty SSMM and siRNA-SSLNP with N/P ratio of 10; (**C**) Empty SSMM and siRNA-SSLNP with N/P ratio of 20; (**D**) Empty SSMM and siRNA-SSLNP with N/P ratio of 30; (**E**) siRNA lipofectamine (siRNA-Lipofectamine) complex; (**F**) Free siRNA molecules.

Lipid-Z (detail on the lipid synthesis is provided under experimental section) was prepared, purified and characterized by reversed-phase high-performance liquid chromatography (RP-HPLC) and matrix-assisted laser desorption/ionization time-of-flight mass spectrometry (MALDI-TOF MS) (Figure S1). Three different formulations of siRNA-SSLNP with nitrogen to phosphate (N/P) ratios (N = arginine amino groups of lipid-Z; P = siRNA phosphate groups) of 30, 20 and 10 were prepared and evaluated to choose the optimum N/P ratio for the preparation. All physicochemical tests were performed in triplicates, and results are expressed as mean ± SD. Particles were found to decrease in size with increasing N/P ratios (Table 1) (Figure 1), which is believed to be a result of greater siRNA condensation with higher nitrogen content. Empty sterically stabilized mixed micelles (SSMM) composed of lipid-Z and DSPE-PEG_2000_ had a constant particle size of ~20 nm. All particles displayed close-to-neutral surface charge as a result of heavy surface PEGylation. siRNA encapsulation efficiency was highest with SSLNP formulation at N/P ratio of 30 with an encapsulation capacity of 4.15 nmol siRNA/0.8 μmol lipid-Z (Table 1) (Figure 1D).

**Figure 2:**
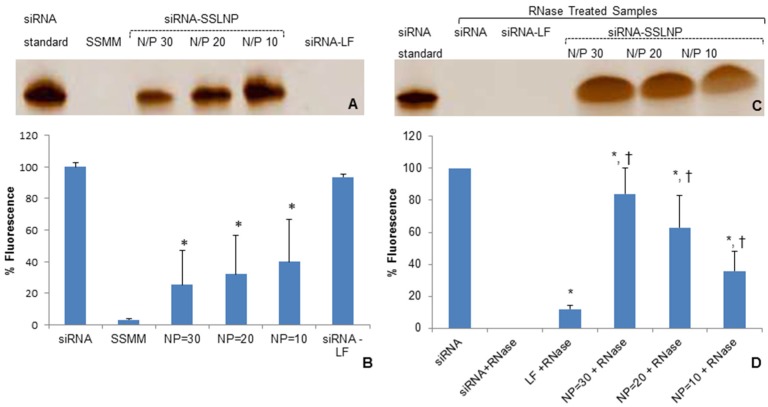
siRNA-SSLNP *in vitro* characterization: (**A**) Gel retardation assay of different formulations of siRNA, containing 200 nM siRNA per sample, on TBE-urea 15% gel , at a voltage of 180 V for 60 min, then stained with 1:500 SYBR Green-II in TBE with mild agitation for 30 min; (**B**) Fluorescence intensities measured by SYBR Green-II exclusion assay of SSMM and siRNA-SSLNP complexes at varying N/P ratios and siRNA with lipofectamine (LF) showing percent of un-incorporated siRNA (* *p* < 0.05 *vs*. free siRNA; mean ± SD; *n* = 3 replicates/group); (**C**) Gel retardation assay of different siRNA formulations after treatment with RNase; (**D**) Fluorescence intensities measured by SYBR Green-II fluorescence assay of different siRNA formulations after treatment with RNase (* *p* < 0.05 *vs*. free siRNA; † *p* < 0.05 *vs.* siRNA-lipofectamine; mean ± SD; *n* = 3 replicates/group).

siRNA-SSLNP formulations with N/P ratios of 10, 20, and 30 were analyzed for their ability to retain siRNA cargo. Freshly prepared test samples underwent gel retardation assay to visually determine presence of uncondensed siRNA (Figure 2A). A separate aliquot of the same test solution was further analyzed by SYBR Green-II exclusion assay to quantify uncomplexed siRNA in the samples; data are expressed as percentage of free siRNA control (Figure 2B). Next, the ability of SSLNPs to protect siRNA against ribonuclease (RNase) enzymatic degradation was evaluated and compared to naked siRNA. In order to quantify entire siRNA content all nanoparticles were disassembled on completion of the enzymatic treatment. As shown in (Figure 2C), after 30 min incubation with RNase I, naked siRNA was completely digested with no band detected; SYBR Green-II assay confirmed the finding with no fluorescence activity detected for free siRNA sample (Figure 2D). In contrast, siRNA band with an intensity close to that of the untreated siRNA control was detected with SSLNP formulation of N/P = 30 and quantified by SYBR Green II exclusion assay (~80% of untreated siRNA) confirming that this preparation has the highest protection potential and therefore was chosen as the optimum formulation.

**Figure 3:**
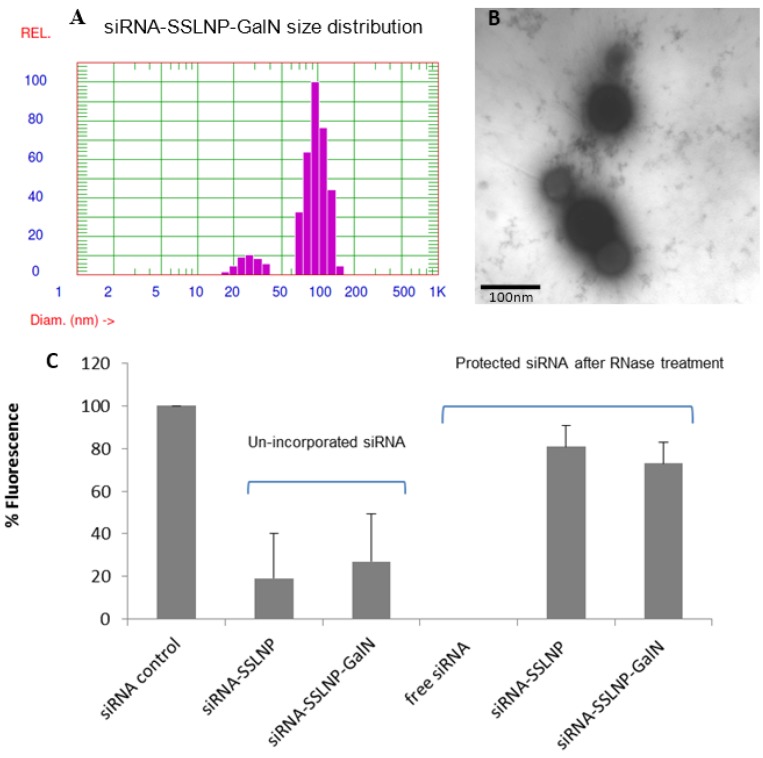
Physicochemical characterization of siRNA-SSLNP-GalN: (**A**) Particle size distribution showing SSLNP-GalN peak at 91 ± 13 nm; (**B**) Transmission electron microscopy (TEM) image of siRNA-SSLNP-GalN, scale bar = 100 nm; (**C**) Results of SYBR Green-II exclusion assay. Bars represent percentage of siRNA before (un-incorporated) and after treatment with RNase enzyme (mean ± SD; *n* = 3 replicates/group).

Particle size and the encapsulation efficiency remained unchanged after GalN surface conjugation (Figure 3A,C). Due to the dynamic properties of the system all measurements were taken in the presence of empty SSMM above its critical micellar concentration (CMC). Hence, two populations of particles were detected both the larger SSLNPs (mean distribution peak ~83 nm) and the smaller SSMM (mean distribution peak ~25 nm). Transmission electron microscopy (TEM) image perfomed using JEM-ARM200F, TEM manufactured by JEOL USA, Inc, Peabody, MA, USA, confirmed the overall shape and average diameter of the final product of siRNA-SSLNP-GaIN (Figure 3B).

**Table 1:** Physicochemical properties of small interfering RNA in sterically stabilized phospholipid nanoparticles (siRNA-SSLNP) compared to sterically stabilized mixed micelles (SSMM) and Small interfering RNA- Lipofectamine (siRNA-Lipofectamine).

Formulation	Particle Size (nm)	Zeta Potential (mV) in Water	siRNA Loading (nmol/mL)	siRNA EE (%)
Free siRNA	2.5 ± 1.3	−42.48 mV	5	--
SSMM	18 ± 2.8	−1.1 mV	--	--
SSLNP (N/P = 10)	98 ± 15	2.07 mV	5	36 ± 12
SSLNP (N/P = 20)	92 ± 13	2.9 mV	5	62 ± 20
SSLNP (N/P = 30)	83 ± 13	6.33 mV	5	85 ± 16
siRNA-Lipofectamine	236 ± 88	30.99 mV	5	--

Values are mean ± SD. EE: encapsulation efficiency.

#### 2.1.2. Transfection Efficiency and Cytotoxicity *in Vitro*

**Figure 4:**
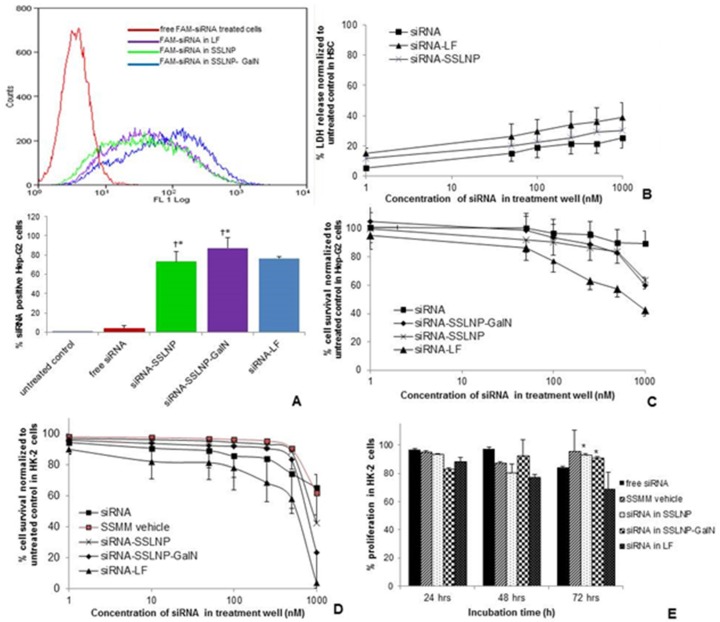
Cell uptake and cytotoxicity assays: (**A**) Hepatic Hep-G2 cell uptake of FAM-labeled siRNA in various complexes. Changes in FACS histogram indicative of siRNA positive cells (upper), bars represent quantitative analysis of FACS histogram as a percentage siRNA-positive cells (lower). (* *p* < 0.05 *vs.* free siRNA and untreated control, † *p* > 0.05 meaning no statistical significance *vs*. siRNA-LF treated cells); (**B**) Cytotoxicity of siRNA in various complexes against primary hepatic stellate cells HSC at different siRNA concentration as determined by membrane integrity (LDH) assay; (**C**) Relative Hep-G2 cell viability expressed as a percentage of untreated control as a measure of cytotoxicity of siRNA complexes using MTS assay after 72 h incubation; (**D**) Cytotoxicity of different siRNA formulations after incubation with renal HK-2 cells for 72 h; (**E**) Cell proliferation kinetics of HK-2 cells after treatment with different formulations at siRNA concentration equivalent to 250 nM assessed at 24, 48, and 72 h time points (* *p* < 0.05 *vs.* siRNA-LF, data on B–D presented as mean ± SD; *n* = 3 replicates/group).

The potential of SSLNP-GalN for siRNA transfection was evaluated *in vitro* on Hep-G2 (human immortalized hepatoma) cells expressing asialoglycoprotein surface receptors [38] in comparison to free siRNA and lipofectamine. Lipofectamine is considered the commercial gold standard for *in vitro* transfection but yet not used for *in vivo* application due to its high cytotoxicity and immunogenicity. The effectivenss of siRNA delivery was measured using fluorescence-activated cell sorting (FACS), to determine the nuber of siRNA positive cells. The flow cytometry histogram of different formulations in (Figure 4A) depicts that all formulations generated significant increment in the mean fluorescence of cells as compared to non-treated cells or free siRNA treated cells. Hep-G2 cells transfected with free FAM-siRNA (FAM or 6-carboxyfluorescein a derivative of fluorescein dye) resulted in 3.4% siRNA positive cells, whereas siRNA in SSLNP and SSLNP-GalN resulted in 73% and 87% siRNA positive cells respectively, which is comparable to results obtained with lipofectamine (LF) 76% (Figure 4A). These results suggest that actively targeted SSLNPs were the most efficient in delivering siRNA into Hep-G2 cells most likely due to receptor mediated endocytosis via interaction between galactosamine and asialoglycoprotein receptors.

Since high positive charge in siRNA formulations can be a concern for its toxicity, we evaluated the cytotoxicity of our siRNA formulations in comparison to free siRNA and siRNA in lipofectamine. First, cell integrity assay (LDH) was performed on cells which are more susceptible to external factors such as primary hepatic stellate cells (HSC). In the range of tested siRNA concentrations (1–1000 nM) during 24 h incubation period lipofectamine formulation possessed slightly higher toxicity, expressed as a percentage of cells with disrupted cell membrane, normalized to untreated control (Figure 4B). SSLNP affected approximately 25% of HSC cell population at siRNA concentration of 200 nM (used for efficacy studies), whereas nearly 35% of cells were impaired by lipofectamine formulation at the same siRNA content. However, variance between the tested groups was not statistically significant.

However, when more robust and proliferative cell lines, such as immortalized hepatic (Hep-G2) and renal tubular epithelial cells (HK-2) were used to evaluate siRNA-SSLNPs toxicity as a measure of cell metabolic activity (MTS assay), results clearly indicated high toxicity of lipofectamine. As shown in (Figure 4C) and after 72 h incubation with test articles the average cell viability observed among Hep-G2 cells incubated with SSLNPs and SSLNP-GalN both containing 200 nM siRNA was around 90%. Hep-G2 cell viability on lipofectamine treatment at the same 200 nM siRNA concentration was only 65% which was significantly different (*p* < 0.05) than siRNA-SSLNP and siRNA-SSLNP-GalN at the same siRNA concentration. Similarly, impact on renal HK-2 cells (Figure 4D) at 200 nM siRNA was similar to the effect observed for the hepatic counterparts: cell viability corresponded to 93% for SSLNP, 90% for SSLNP-GalN, and 68% for lipofectamine. However, renal HK-2 cells were greatly affected by all tested formulations at a high siRNA concentration of 1000 nM, (Figure 4D) which was not as dramatic in hepatic Hep-G2 cells (Figure 4C). An intense suppression of the cell proliferation was observed for siRNA-lipofectamine that resulted in near complete loss of viable cells (3.5% of control). When comparing the proliferation kinetics of different formulations with preparations containing 250 nM siRNA, we were able to show significantly less cytotoxicity of the SSLNP compared to lipofectamine at 72 h (Figure 4E).

#### 2.1.3. Protein Downregulation *in Vitro*

**Figure 5:**
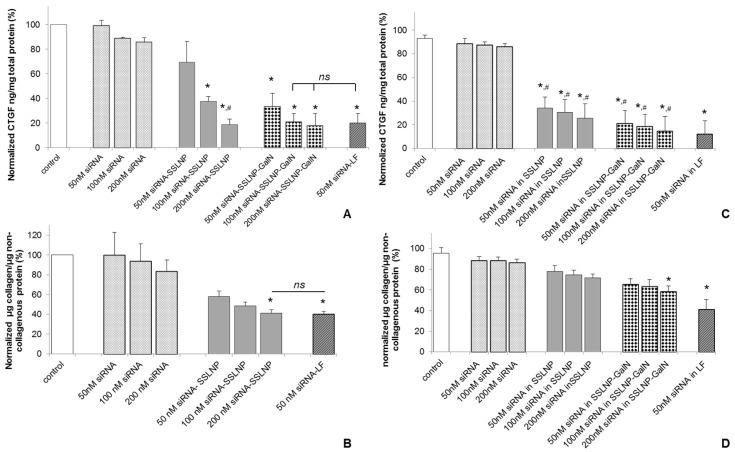
Protein downregulation: (**A**) Reduction of connective tissue growth factor (CTGF) expression in human hepatic Hep-G2 cells 24 h post transfection with anti CTGF-siRNA in different complexes; (**B**) Reduction of extracellular matrix (ECM) collagen expression in primary human hepatic stellate cells (HSC) 24 h post transfection with anti CTGF-siRNA in different complexes; (**C**) GTGF protein downregulation in renal tubular HK-2 cells, activated with transforming growth factor β1 (TGF-β1), 72 h post transfection with anti CTGF-siRNA in different complexes; (**D**) Reduction in ECM collagen expression by TGFβ activated HK-2 cells 72 h post transfection with anti CTFG-siRNA in different complexes. (Data are expressed as percent of the untreated control; mean ± SD; *n* = 3/treatment; * *p* < 0.05 *vs*. free siRNA at a corresponding siRNA dose; ^#^ *p* > 0.05 or statistically not significant *vs.* siRNA-lipofectamine (LF); ns—non significant among the groups indicated). The scrambled siRNA treatment showed no significance as compared to untreated controls and therefore control refers to untreated controls (Data not plotted).

These studies were carried out using two test parameters; one by measuring the CTGF protein levels in target cells and second; by studying the reversal of the activation of myofibroblasts which participate in fibrosis. Since attachment of galactosamine to a variety of uncharged polymers can facilitate targeting hepatocytes via asialoglycoprotein receptor interaction [33,39] galactosamine targeting can enhance endocytosis of SSLNP, and increase the amount of siRNA-SSLNP in the cells. This would enable greater gene silencing. CTGF protein expression was measured to evaluate gene silencing potencies of different siRNA formulations in hepatic Hep-G2 and renal HK-2 cells (activated by TGF-β1). These cells were used as models for hepatocytes and renal tubular epithelial cells known to express high amounts of CTGF protein *in vivo* during fibrosis. Cells were treated with various siRNA-SSLNP formulations at different concentrations or siRNA in lipofectamine as a positive control. As shown in (Figure 5A) a reduction of about 85% in CTGF protein expression was observed in Hep-G2 in comparison with the untreated control. Although a slight difference was observed with the targeted and non-targeted formulations in downregulating CTGF expression, it was not significantly different at siRNA conc. of 200 nM, *p* > 0.05. The results of our targeted formulation SSLNP-GalN loaded with 100 and 200 nM siRNA were comparable to that of lipofectamine loaded with 50 nM siRNA. Meanwhile HK-2 cells expression of CTGF (Figure 5C) decreased to around 20% in cells treated with 50 nM siRNA in SSLNP-GalN. Free siRNA resulted in only a minimal reduction in CTGF expression in both cell lines. These results indicated that the gene silencing effect was due to the enhanced stability and uptake of siRNA when incorporated within SSLNPs conjugated with galactosamine. Interestingly, in hepatocytes (Hep-G2) there was no dose dependent response observed at 100 and 200 nM siRNA in SSLNP-GalN (Figure 5B) which may be explained by asialoglycoprotein receptor saturation. Whereas, with renal cells (HK-2) reduction of CTGF expression was not significantly different in targeted *versus* non targeted siRNA nanomedicine (Figure 5C), which may be a result of lower expression of asialoglycoprotein receptors on these cells.

To further confirm the expected effect on downregulation of CTGF, we tested the ability of our siRNA nanomedicine to reverse the activation of myofibroblasts, an effect that is expected to take place *in vivo* upon CTGF knockdown by intracellular delivered siRNA. As mentioned earlier, the down-modulation of CTGF activity is expected to shift the TGF-β/BMP-7 balance in the direction of anti-fibrosis [25], *i.e.*, inhibiting ECM synthesis, epithelial–mesenchymal transition and HSC-activation, and increasing ECM-degradation. Therefore, we also assessed the anti-fibrotic effect of anti-CTGF siRNA by measuring the amount of collagen deposits in the ECM of cells possessing myofibroblast phenotype in the cell culture conditions, namely HSC cells cultured on flat surface and TGF-β1 activated renal HK-2 cells (Figure 5B,D). Collagen expression was measured using Sirius red/fast green kit after the treatment of activated HSC with different siRNA concentrations in SSLNPs or lipofectamine as a positive control. As shown in (Figure 5B), in hepatic stellate cells the reduction in collagen expression followed a dose dependent response. The highest reduction of 52% was observed in the ECM of cells treated with 200 nM siRNA in SSLNP. This result was not significantly different from that of lipofectamine. Galactosamine conjugated SSLNPs were not used in this treatment since activated myofibroblasts are not known to express asialoglycoprotein receptors. The collagen expression reduction followed a dose dependent trend with HK-2 cells (Figure 5D) treated with siRNA-SSLNP, showing a total collagen expression of 74% at 100 nM CTGF-siRNA. Galactosamine targeted formulation at 200 nM dose showed better results in terms of ECM degradation with up to 50% reduction in total collagen compared to untreated control (untreated HK-2 cells).

**Figure 6:**
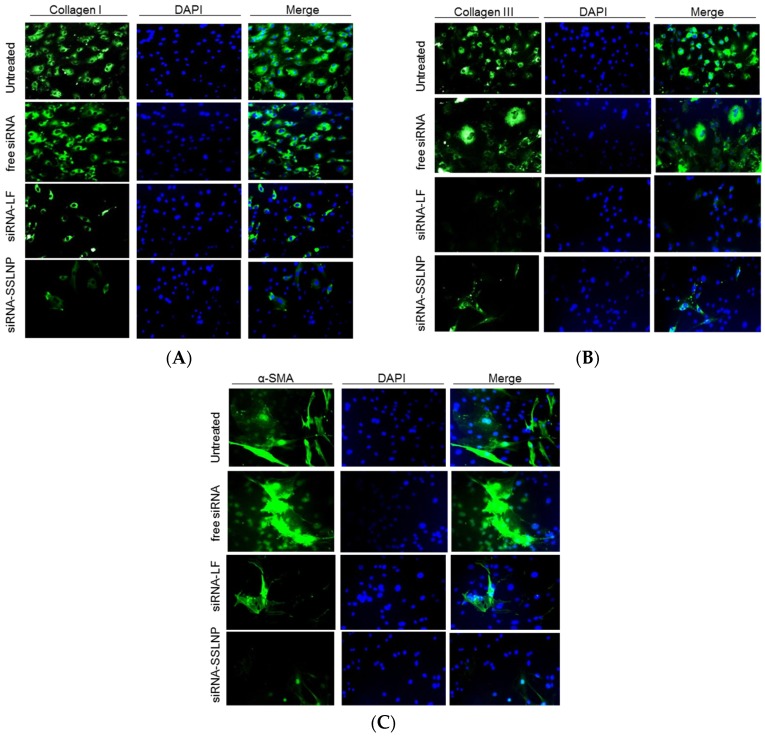
Reversal of primary hepatic stellate cell (HSC) activation: (**A**) Down-regulation of collagen I; (**B**) Collagen III; and (**C**) α-smooth muscle actin (α-SMA) protein expression indicative of the reversal of activated myofibroblasts to quiescent stellate cells. Activated HSC were transfected with connective tissue growth factor (CTGF-siRNA) in various formulations. Standard immunocytochemistry performed 24 h post-transfection, cells were probed with primary antibodies followed by secondary Alexa-Fluor 488 (**green**) labeled antibody, then DAPI for nuclear staining (**blue**).

We then confirmed the above results, by performing immunocytochemistry for α-smooth muscle actin (α-SMA) and Collagen type I and type III in Hepatic stellate cells (HSC) cultured on glass slides to confirm the reversal of their activation. A marked decline was observed in the expression of these proteins in cells treated with siRNA formulated with SSLNP or lipofectamine in comparison with free siRNA and untreated control (Figure 6). Collectively, these results indicate the reversal of HSC activation to their quiescent phenotype.

#### 2.1.4. *In Vivo* Bio-Distribution and Pharmacokinetics

**Figure 7:**
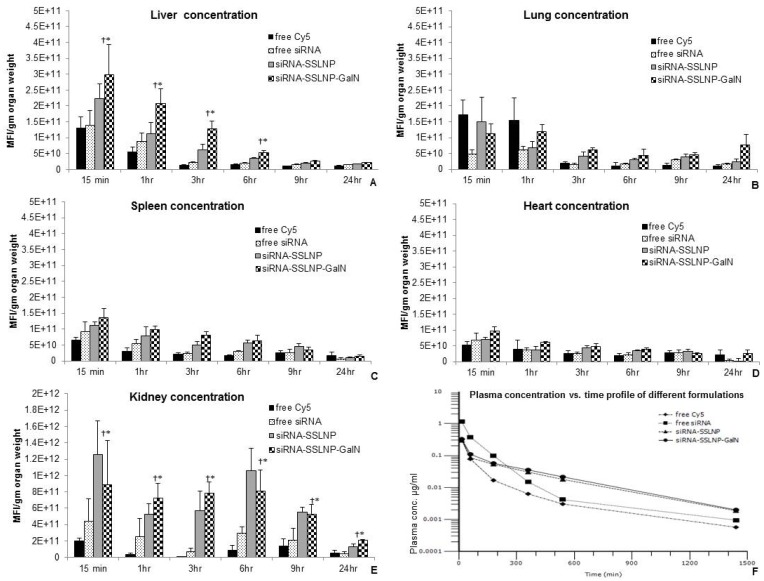
Biodistribution of different siRNA formulations compared to free Cy5 fluorophore over 24 h periods in (**A**) liver; (**B**) lung; (**C**) spleen; (**D**) heart; and (**E**) kidneys. Targeted formulation (siRNA-SSLNP-GalN) shows significant concentrations in liver and kidneys over observation period (*n* = 4 for each time point; * *p* < 0.05 *vs.* free siRNA treated animals, † *p* < 0.05 *vs.* free Cy5 treated animals); (**F**) Plasma concentration *vs.* time after single intravenous administration of various Cy-5 labeled formulations in Balb/c mice. (Data are presented as mean ± SD; *n* = 4 animals/each time point, MFI-Mean fluorescence intensity).

We investigated the *in vivo* bio-distribution (BD) and pharmacokinetics (PK) of our SSLNP nano-construct to confirm its targetability to hepatic or renal tissues. Organ uptake and PK parameters were evaluated in healthy 6 week old male balb/c mice *in vivo*. Formulations containing, either free Cy5, and Cy5-labelled siRNA formulations were administered through tail vein, and animals were sacrificed at predetermined time points over a 24 h period. (Figure 7) displays the distribution of the drug based on the intensity of Cy5 fluorescence indicating amounts of the tested formulations in different organs as well as plasma concentration at various post-injection time points. Higher amounts of siRNA were delivered to the liver and kidneys compared to other tissues while significantly less amounts were delivered when siRNA was administered in its free form. Furthermore, with the actively targeted formulation siRNA amounts in the liver was higher than the passively targeted formulation. However, in the kidneys the active targeting was not as prominent but was always higher compared to free siRNA. (Figure 7A,E). Furthermore, kidney targetability demonstrated to be more efficient. These results indicate the advantage of using SSLNP nanocarrier for passive targeting and specifically SSLNP-GalN for active hepatic and renal targeting. In this context, passive targeting refers to spontaneous uptake of SSLNPs by renal cells *vs.* SSLNP-GalN intended for active targeting. The results in (Figure 7) showing similar or slightly higher kidney concentration of SSLNPs compared to SSLNP-GalN indicates that passive uptake plays a greater role in the kidney unlike in liver tissue where active targeting noticeably complements the passive targeting.

**Table 2:** Pharmacokinetic parameters of different siRNA formulations.

Formulation	A (g/mL)	B (μg/mL)	α (min^−1^)	β (min^−1^)	Vp (mL)	V_dss_ (mL)	t_½, α_ (min)	t_½, β_ (min)	AUC (μg·min/mL)	AUMC (μg·min^2^/mL)	MRT (min)	Cl (L/min)
Free Cy5	0.54	0.04	0.04	0.005	1.03	2.52	17.18	138.26	21.44	1933	91.17	0.028
Free Cy5 labeled siRNA	1.51	0.063	0.06	0.0010	0.28	0.51	11.27	67.39	85.73	6353	74.10	0.006
Cy5-siRNA in SSLNP	0.60	0.09	0.07	0.0030	0.86	4.01	0.07	228.37	38.79	10,068	259.56	0.015
Cy5-siRNA in SSLNP-GalN	0.46	0.09	0.04	0.0027	1.08	3.91	0.045	258.11	44.71	13,052	291.86	0.013

(A) residual phase y-intercept, (B) elimination phase y-intercept, (α) distribution phase rate constant, (β) elimination phase rate constant, (Vp) volume of central compartment, (V_dss_) apparent volume of distribution, (t_½, β_) elimination half-life, (t_½, α_) distribution half-life, (AUC) area under plasma concentration *vs.* time curve, (AUMC) area under moment curve, (MRT) mean residence time, (Cl) clearance. Data are mean values from pooled analyses.

Pharmacokinetic parameters displayed in (Table 2) were calculated according to two-compartmental model analysis. The elimination phase of the non-targeted (SSLNP) and targeted (SSLN-GalN) nanoparticles was significantly prolonged (*p* < 0.05) with half-lives of 228 min (3.8 h) and 258 min (4.6 h) respectively in comparison to 67 min (~1 h) observed with free siRNA. Additionally, they showed significantly higher volume of distribution at steady state (*p* < 0.05) with values of 4.0 mL for SSLNP and 3.9 mL for SSLNP-GalN compared to 0.5 mL for the free siRNA formulation. This high volume of distribution can be credited to the larger tissue uptake of the nanoparticels, while the charged free siRNA had higher plasma protein binding affinity resulting in its delayed tissue distribution and elimination.

Interestingly, both targeted and non-targeted formulations demonstrated high plasma clearance rates, 0.015 mL/min and 0.013 mL/min for SSLNP and SSLNP-GalN respectively, which were about 8 folds faster than plasma clearance of free siRNA (0.006 mL/min), while their mean residence times (MRT) were significantly higher (*p* < 0.05), with values of 260 min and 292 min respectively, than that of free siRNA (74 min). This observation can be explained by the fast uptake and longer retention of the nanomedicine in organs (mostly liver and kidney). Finally, values calculated for AUMC appear to be significantly higher with the use of nanoparticles (*p* < 0.05), suggesting higher organ distribution of siRNA-SSLNP and siRNA-SSLNP-GalN in comparison to free siRNA, although calculated AUC values did not support this finding and requires further investigation.

### 2.2. Discussion

RNAi therapeutics represents an emerging modality for the treatment of many life threatening diseases [15,40]. Due to its low serum stability, development of a safe and effective *in vivo* delivery system is of paramount importance to achieve optimal effectiveness of RNAi therapeutics [1]. Numerous strategies including viral and non-viral delivery systems have been studied to achieve effective delivery of siRNA *in vivo* [41], with non-viral options being the safer alternative for their lower immunogenicity and toxicity, yet RNAi therapeutics remain an unmet medical need with no FDA approved product in market.

In this study, we have developed, characterized and evaluated SSLNP as a nanocarrier for the passive and active targeted delivery of siRNA *in vivo*. This nanocarrier is the first of its kind to use relatively inexpensive, naturally occuring and biocompatible molecule components for siRNA delivery. SSLNP formulation developed in this study is based on the novel Lipid-Z composed of phospholipid monomer and arginine amino acids. The four arginine head group of lipid-Z carries four positive charges in physiological pH of 7.4, the complexion of lipid-Z with DSPE-PEG_2000_ in our formulation decreases the available positive groups that can interact with siRNA; hence, it was important to optimize the amount of lipid-Z needed for efficient encapsulation of siRNA. N/P ratio of 30 was sufficient to permit the entrapment of 4.15 nmol siRNA/0.8 μmol lipid with an overall particle size below 100 nm and a slight positive charge (Table 1). This optimum ratio was chosen by taking into account encapsulation efficiency, particle size and protection against enzymatic degradation.

As surface PEGylation have been reported to compromise the transfection efficacy of nanoparticles [42], we used a surface conjugated ligand galactosamine to target and SSLNPs internalization into cells of interest through receptor mediated endocytosis. Galactosamine was successfully conjugated to the surface of siRNA-SSLNP without affecting the physicochemical properties of nanoparticles (Figure 3). The ability of SSLNP-GalN to deliver siRNA into cells was evaluated on hepatic Hep-G2 cells with FAM-labeled siRNA and showed comparable transfection efficiency, yet significantly lower cytotoxicity than lipofectamine the commercial gold standard for *in vitro* transfection (Figure 4).

It can be assumed that once the particles are taken up by the cells and trafficked to endosomes, the acidic environment promotes the release of siRNA from SSLNPs, this could be attributed to the fusion of the nanocarriers phospholipids with the endosomal bilayer [43]. The cytosol released siRNA cargo maintained its biological activity by binding to CTGF-mRNA and causing sequence-specific degradation of CTGF-mRNA, this can be inferred from the significant downregulation of CTGF protein expression in cells treated with siRNA-SSLNPs in comparison to free siRNA control (Figure 5) as well as the reversal of activation of HSC and fibrolysis of ECM (Figure 5 and Figure 6). Taken together, the uptake and silencing data indicate the importance of vehicle stability for targeted delivery to the final destination and silencing over-expressed proteins.

As mentioned earlier, the developed nanoparticles are intended for the passive and active delivery of siRNA, which is inversely proportional to the time and amount of siRNA in circulation. The nano size of our particles allows their extravasation from liver sinusoids and glomerular endothelial fenestrations (100–150 nm diameter) [42,44] giving them direct access to hepatocytes and renal tubular epithelial cells. Meanwhile, galactosamine targeting enhances their uptake by renal tubular epithelial cells and hepatocytes through receptor mediated interaction. Based on our results, higher amounts of SSLNP-GalN encapsulated siRNA were retained in the liver and kidneys for a longer duration of time, compared to free siRNA (Figure 7). However, further studies are required to evaluate the bio-distribution of siRNA in SSLNP-GalN in fibrotic animals, to identify if the interstitial collagenous structure of hepatic and renal fibrosis affects the organ uptake of siRNA and to evaluate efficacy of this nanomedicine *in vivo* in the reversal of fibrosis.

## 3. Experimental Section

### 3.1. Materials

1,2-Distearoyl-*sn*-glycero-3-phosphatidylethanolamine-N-[methoxy(polyethyleneglycol)-2000] sodium salt (DSPE-PEG_2000_) was purchased from Lipoid GmbH (Ludwigshafen, Germany). 1,2-Dipalmitoyl-*sn*-Glycero-3-Phosphothioethanol Sodium Salt (Ptd Thioethanol) and 1,2-distearoyl-*sn*-glycero-3-phosphoethanolamine-*N*-[carboxy(polyethyleneglycol)-2000] ammonium salt (DSPE-PEG_2000_ Carboxylic Acid) were from Avanti polar lipids, Inc. (Alabaster, AL, USA). d-galactosamine hydrochloride and *N*-(3-dimethylaminopropyl)-*N*ʹ-ethylcarbodiimide (EDC) were from Thermo Fisher Scientific (Pittsburgh, PA, USA). *N*-hydroxysuccinimide (NHS), negative siRNA control and Cy5-labeled siRNA were purchased from Sigma-Aldrich (St. Louis, MO, USA). siRNA against CTGF was obtained from Santa Cruz biotechnology (Dallas, TX, USA). RNase One Ribonuclease, CellTiter-96 AQ-one Solution Cell proliferation Assay and CytoTox-one homogeneous membrane integrity assay were purchased from Promega, Inc. (Madison, WI, USA). FAM-labeled siRNA, lipofectamine, SYBR Green-II and Alexa-fluoro 488 donkey-anti-rabbit IgG secondary antibody were from Invitrogen™ Life Technologies (Grand Island, NY, USA). Trypsin-EDTA (0.25% with 0.53 mM EDTA), Minimum essential media (MEM), fetal bovine serum (FBS), non-essential amino acids, antibiotic solution (penicillin 10,000 units/mL with streptomycin 10 mg/mL) and sodium pyruvate were all purchased from Mediatech-Cellgro (Manassas, VA, USA). Keratinocyte serum free medium and supplements (K-SFM) were purchased from Invitrogen™ Life Technologies (Grand Island, NY, USA). Human hepatocellular carcinoma cells (Hep-G2) and immortalized proximal renal tubular epithelial cells (HK-2) were obtained from the American Type Culture Collection (Manassas, VA, USA). CTGF-ELISA kit was from Antigenix, Inc. (Huntington Station, NY, USA). Human primary hepatic stellate cells (HSC) as well as corresponding stellate cell media (SteCM) and supplements were from Sciencell (Carlsbad, CA, USA). Sirius red/fast green kit was from Chondrex, Inc. (Redmond, WA, USA). Primary anti-collagen I, anti-collagen-III and anti-α-SMA antibodies were from Abcam (Cambridge, MA, USA). Six-week old male Balb/c mice were obtained from Harlan Laboratories (Indianapolis, IN, USA). Other materials, if not specified, were purchased from Thermo Fisher Scientific (Pittsburgh, PA, USA) or Sigma-Aldrich (St. Louis, MO, USA).

### 3.2. Cationic Lipid-Z Synthesis

Four arginine (4R) peptide synthesis was performed by solid phase peptide synthesis method using Fmoc-AA-Wang resin (50 µmole) and Symphony^®^ Peptide Synthesizer (Protein Technologies Inc, Tucson, AZ, USA). Peptide was synthesized using cycles that started with the removal of Fmoc group, using 20% piperidine in *N*,*N*-Dimethylformamide (DMF) (2 × 5 min) followed by washing the resin with DMF (6 × 30 s). The first amino acid (Fmoc protected, 2 equivalent) was added in the presence of 0.4 M *O*-Benzotriazole-*N*,*N*,*N*ʹ,*N*ʹ-tetramethyl-uronium-hexafluoro-phosphate (HBTU, 1.9 equivalent), and 0.8 M *4-*methylmorpholine (NMM, 4 equivalent) in DMF (3 × 30 min), amino acids were added in cycles. Excess reagents were washed (6 × 30 s) with DMF. The synthesis took place from *C*-terminal to N-terminal; amino acids side groups were protected during the synthesis.

For the coupling of 4R peptide to phospholipid, resin was washed with 0.5% *N*,*N*-Di isopropylethylamine (DIEA) in DMF (5 × 1 mL). *m*-maleimidobenzoyl-*N*-hydroxysuccinimide ester (MBS, 1.1 equivalent) and DIEA (1.1 equivalent) in 1 mL DMF were added to the resin and stirred for 2 h at room temperature. Second coupling was done with the same amounts of reagents, stirred at 4 °C, overnight. Resin was then washed with DMF (5 × 1 mL). Ptd Thioethanol Lipid (1.1 equivalent) was dissolved in chloroform and was added to the resin along with 1.1 equiv of DIEA. The reaction was run for several hours at room temperature. A second coupling was done with the lipid to ensure the reaction has gone to completion. The resin was then washed with DMF (5 × 1 mL), methylene chloride (5 × 1 mL) and dried. The conjugated peptide was cleaved from resin with 100% Trifluoroacetic acid (TFA) for 1.5 h and product was purified by reversed phase high performance liquid chromatography (RP-HPLC) on a Vydac™ protein and peptide C18 column. The final product was then identified by matrix-assisted laser desorption/ionization time-of-flight mass spectrometry (MALDI-TOF MS) (Figure S1).

### 3.3. Galactosamine-DSPE-PEG_2000_ Coupling

DSPE-PEG_2000_-COOH (1 equiv) was activated by the reaction with EDC (10 equivalent) in 2 mL dimethyl sulfoxide (DMSO) for 2 h at room temperature. NHS (10 equivalent) was then added to the mixture and stirred overnight at room temperature. d-galactosamine HCl (2 equiv) was reacted with triethylamine (2 equivalent) overnight at room temperature to produce the free base. d-galactosamine base was then added to the activated DSPE-PEG_2000_-COOH and the reaction was run with continuous stirring in the dark at room temperature for 48 h. The resulting solution was then dialyzed using pre-treated regenerated cellulose (RC) Spectra/Por 7 dialysis membrane with molecular weight cut off size (MwCO) of 1000 Da (Spectrum Laboratories, Inc., Rancho Dominguez, CA, USA) against phosphate buffered saline (PBS) for 24 h, then against distilled water for another 24 h (to remove unreacted galactosamine, coupling reagents and DMSO). The resulting solution was lyophilized using the LabconcoFreeZone^®^ 6 L FreezeDry System (Labconco, Kansas, MO, USA). The obtained powder was evaluated for successful conjugation using MALDI-TOF MS (Figure S2) and nuclear magnetic resonance (NMR) spectroscopy (Figure S3).

### 3.4. Preparation of siRNA-SSLNP Complexes

siRNA-SSLNP complexes were prepared by film rehydration method [45] with different nitrogen to phosphate (N/P) ratios (30, 20 and 10). Briefly, Lipid-Z and DSPE-PEG_2000_ were dissolved separately in methanol then mixed in round bottom flasks at appropriate ratios. The solvent was subsequently removed using a vacuum rotary evaporator (BUCHI Labortechnik AG; Flawil, Switzerland) under a stream of argon and vacuum (600 mm Hg pressure) at 50 °C and 150 rpm for 30 min. The residual solvent from the resulting film was removed under vacuum overnight in dark. Thereafter, the dried film was rehydrated with 5 nmol of siRNA in nuclease free water. The resulting dispersion was vortexed until the film was dissolved, followed by bath sonication for 5 min. Flasks were then flushed with argon, sealed, and allowed to equilibrate in the dark for 2 h at 37 °C with continuous stirring to produce siRNA-SSLNP. Samples were then extruded through Nylon membranes with pore sizes of 200, 100 and 50 nm to ensure uniformity and particle size of <100 nm. Previously, we showed with an aid of isothermal titration calorimetry that insertion of ligand-conjugated lipid to pre-formed particles via self-association has a surface saturation point of approximately 5.6% without significant changes in particle properties [46]. Based on this data we aimed for 10% galactosamine conjugation taking into account for the smaller size of GalN as compared to the ligand used in the previous study. Accordingly, appropriate amounts of DSPE-PEG_2000_-GalN was incubated with the preformed particles and allowed to self-associate for 2 h. Empty SSMM were prepared using equal ratios of Lipid-Z and DSPE-PEG_2000_ following the same procedure described above and reconstituted with siRNA-free nuclease-free water.

### 3.5. Physicochemical Characterization

Particle size distribution and zeta potential of the prepared samples were measured using dynamic light scattering (DLS) and electrophoretic light scattering (ELS) respectively by the particle sizer (Agilent 7030 NICOMP DLS/ZLS, Santa Clara, CA, USA) equipped with a 100 mW He-Ne laser (632.8 nm excitation wavelength) and set up at a fixed scattering angle of 90°. Solvent viscosity and refractive index of water were used with values of 0.933 cP, and 1.333 respectively. Samples were measured at room temperature and 1 atm pressure. The mean hydrodynamic particle diameters (***d**_h_*) of particles in the aqueous dispersion, were calcaulated according to the Stokes-Einstein equation using the measured diffusion of particles in solution, while zeta potential (ζ) was determined using the Smoluchowski approximation. The reported experimental results were the average of at least three values obtained from analysis of the autocorrelation function accumulated for at least 15 min.

Transmission electron microscopy (TEM) images of the prepared siRNA-SSLNP and siRNA-SSLNP-GalN were acquired using a JOEL manufactured JEM-1220 transmission electron microscope (JEOL USA, Inc, Peabody, MA, USA) fitted with a tungsten electron source. Briefly, freshly prepared siRNA-SSLNP complexes (5 μL) were spotted onto 300-mesh format carbon-coated grids (Electron Microscopy Sciences, Hatfield, PA) and incubated for 5 min at room temperature. Negative staining was performed with 0.5% uranyl acetate (40 μL). Samples were air-dried. All TEM images were acquired at an accelerating voltage of 80 kV by GatanEs1000W 11MP CCD camera. Digital Micrograph software was used to analyze the resulting images.

For gel retardation studies, samples containing 200 ng of siRNA, with varying N/P ratios in nuclease free water, were electrophoresed through 15% Novex TBE-urea gel (Invitrogen™-life technologies, Grand Island, NY, USA) in TBE running buffer. Gels were run at a voltage of 180V for 60 min, then stained with 1:5000 SYBR Green-II in TBE with mild agitation for 30 min, after which they were photographed under UV light using BioRad Gel-Doc imaging system (Life Science Research, Hercules, CA, USA).

SYBR Green-II exclusion assay was performed to quantify the encapsulation of siRNA within SSLNP using the fluorescence quenching method. These experiments were carried out by measuring the fluorescence intensity of siRNA-SSLNP complexes, prepared with different N/P ratios, as a result of the intercalation between siRNA and SYBR Green-II. Fluorescence was measured using 96-well plate reader BioTek Synergy 4, manufactured by BioTek (Winooski, VT, USA) at excitation and emission wave lengths of 497 nm and at 520 nm respectively. Percentage of encapsulated siRNA was determined from the relative fluorescence obtained with each sample to that of SYBR Green-II and siRNA in the absence of lipids.

Nuclease resistance of SSLNP incorporated siRNA was determined after the treatment of samples with 1U of RNase I ribonuclease/µg siRNA for 30 min at 37 °C. 0.1% Triton-X 100 was used to terminate RNase activity and Heparin sodium 50 U/µg siRNA was used to disassemble SSLNPs nanoparticles. Gel retardation technique and SYBR Green-II exclusion assay were repeated to determine the integrity of the preserved SSLNP siRNA compared to free siRNA.

### 3.6. Cytotoxicity and Cell Uptake Studies

Hepatic Hep-G2 cells were seeded at a density of 6000 cells/well in 96-well plates and allowed to attach for 24 h at 37 °C and 5% CO_2_. Hep-G2 cells were cultured in 100 µL/well MEM medium, containing 10% FBS, 1 mM sodium pyruvate, 1% nonessential aminoacids, 100 U/mL penicillin, and 100 µg/mL streptomycin. After cell attachment, the medium was replaced with 100 µL/well fresh complete media, containing serial dilutions of vectors with a molar siRNA concentration ranging from 1 to 1000 nM, and incubated for 72 h. At the end of the incubation period, Hep-G2 were treated with CellTiter-96 AQ-one solution cell proliferation (MTS) assay according to manufacturer’s instructions. Cell viability in each well was determined by absorbance of the formazan product recorded at 490 nm by a BioTek Synergy 4 plate reader (Winooski, VT, USA) normalized to untreated control.

Primary HSC were seeded in 96-well plates at a density of 6000 cells/well and allowed to attach for 24 h at 37 °C and 5% CO_2_. HSC were cultured in 100 µL/well Stellate Cell Medium (SteSM), supplemented with 10% FBS, stellate cell growth supplements, and 100 U/mL penicillin, and 100 µg/mL streptomycin. After cell attachment, the medium was replaced with FBS free medium containing serial dilutions of vectors with a molar siRNA concentration from 1 to 1000 nM, and incubated overnight (16 h). At the end of the incubation period cells were subjected to CytoTox-one homogeneous membrane integrity (LDH) assay according to manufacturer’s protocols. Cell integrity was assessed by fluorometry at an excitation wavelength of 560 nm and an emission of 590 nm using BioTek Synergy 4 plate reader, manufactured by BioTek (Winooski, VT, USA). Data were normalized to untreated controls.

To assess the effect of SSLNPs on the proliferation of renal HK-2 cells over an incubation period of 72 h, cells were seeded at a density of 5000 cells/well in 96-well plates and allowed to attach for 24 h at 37 °C and 5% CO_2_. Cells were cultured in Keratinocyte Serum Free Medium supplemented with 0.05 mg/mL bovine pituitary extract (BPE), 5 ng/mL human recombinant epidermal growth factor (EGF) and antibiotic solution (penicillin 10,000 units/mL with streptomycin 10 mg/mL). After overnight attachment, medium was replaced with 100 μL/well fresh media containing serial dilutions of the following formulations, with a molar siRNA concentration ranging from 1 to 1000 nM: free scrambled-siRNA, scrambled -siRNA in SSLNP (with N/P ratios of 30), scrambled-siRNA in SSLNP-GalN or scrambled-siRNA in lipofectamine (LF). In addition, HK-2 cells were also incubated for 24, 48 or 72 h at the same incubation conditions with various siRNA formulations at a set siRNA concentration of 250 nM. At the end of incubation periods MTS solution was added to wells and plates were further incubated in the dark for 3 h after which the absorbance of formazan was measured at 490 nm using a BioTek Synergy 4 plate reader (Winooski, VT, USA). The results were normalized to untreated control and percentage of cell viability was calculated per treatment.

To assess the ability of SSLNP and SSLNP-GalN to transfect siRNA into cells in comparison to lipofectamine (LF), carboxyfluorescein (FAM)-labeled siRNA was formulated in SSLNPs at N/P ratio of 30 as described above. Hep-G2 cells were seeded in 6-well plates at a density of 200,000 cells/well as described above and incubated for 24 h prior to transfection. Cells were treated with either free FAM-siRNA, siRNA-SSLNP, siRNA-SSLNP-GalN at siRNA concentration of 200 nM or siRNA-LF at 50 nM concentration according to manufacture recommendations. Treated cells were incubated overnight then washed with PBS and trypsinized. The uptake of FAM-siRNA mediated with different vectors was detected using Beckman Coulter Cyan ADP flow cytometry and analyzer, manufactured by Beckman (Indianapolis, IN, USA).

### 3.7. In Vitro Protein Downregulation

To evaluate CTGF downregulation, hepatic Hep-G2 cells and renal HK-2 cells were seeded in 24-well plates at a density of 50,000 cells/well at conditions described above and incubated for 24 h prior to treatment. CTGF-siRNA complexes with SSLNP and SSLNP-GalN were prepared at N/P ratio of 30 as previously described. Cells were treated with either free CTGF-siRNA, CTGF-siRNA in SSLNP, or CTGF-siRNA in SSLNP-GalN at siRNA concentrations of 50, 100 and 200 nM, while positive control cells were treated with CTGF-siRNA in LF at 50 nM concentration according to manufacture recommendations. Treated cells were incubated overnight then analyzed for CTGF expression 24 h post-transfection using CTGF-ELISA kit (Antigenix, Inc., Huntington Station, NY, USA) according to the manufacture’s protocol. Results were normalized to total protein in samples measured by Bradford protein assay.

For the evaluation of collagen type I and III as well as α-SMA expression immunocytochemistry technique was used. Primary HSC were seeded at a density of 5000 cells/well on German glass slide with cover in 0.25 mL SteCM/well as described above. 24 h after incubation, cells were treated with either free CTGF-siRNA or CTGF-siRNA in SSLNP at siRNA concentration of 200 nM, while positive control cells were treated with CTGF-siRNA in LF at 50 nM siRNA concentration and incubated overnight. Cells were then washed three times with (37 °C) PBS with Ca^2+^ and Mg^2+^ and fixed in ice-cold methanol for 10 min, washed three times with PBST (0.1% tween in PBS), and incubated in PBST containing 1% bovine serum albumin (BSA) for 30 min. All primary antibodies (anti-collagen I, anti-collagen-III and anti-α-SMA) incubations were performed overnight at 4 °C in 1% BSA in PBST. Following three PBST washes, cells were incubated with Alexa-Fluor 488 conjugated secondary donkey-anti-rabbit antibody in 1% BSA in PBST for 1 h at room temperature and followed by three washes with PBST. Cell nuclei were stained with DAPI (4,6-diamidino-2-phenylindole) included in Vectashield mounting media (Vector Laboratories, Burlingame, CA, USA). Images were acquired using an Olympus IX70 inverted fluorescence microscope, manufactured by Olympus microscopy, Pennsylvania, USA. coupled with a QImaging RETIGA 1300 cooled-CCD digital camera; and processed using QCapture Pro™ 6 software.

To measure the amount of collagen deposit in the extracellular matrix of HSC, cells were seeded in 24-well plate at a density of 50,000 cells/well 24 h prior to treatment as described above. Cells were treated with either free CTGF-siRNA, CTGF-siRNA in SSLNP, or CTGF-siRNA in SSLNP-GalN at siRNA concentrations of 50, 100 and 200 nM, while positive control cells were treated with CTGF-siRNA in LF at 50 nM concentration and incubated overnight. Cells were then washed three times with (37 °C) PBS with Ca^2+^ and Mg^2+^ and fixed in ice-cold ethanol for 10 min, then dyed with Sirius red/fast green (Chondrex, Inc., Redmond, WA, USA) according to the supplier’s protocol. Briefly, dye solution was added and plates incubated for 30 min at room temperature. Dye solution was then removed by washing for multiple times until fluid appeared colorless. Dye extraction solution, provided with kit, was added to each well and mixed gently until color eluted from cells and ECM. Absorbance was measured using a BioTek Synergy 4 plate reader at 540 nm and 605 nm from which total collagen was calculated and normalized to total non-collagenous protein in well using the following formulas according to supplier’s protocol:

- Collagen (µg/well) = (OD 540 − (OD 605 × 0.291)/37.8 × 1000
- Non-Collagen Protein (µg/well) = OD 605/2.04 × 1000

Renal HK-2 cells were seeded in 12-well plate at a density of 100,000 cells/well and allowed to attach and activate into myofibroblasts with 3 ng/mL TGF-β1 for 48 h at 37 °C and 5% CO_2_ [47]. The cells were incubated with 1 mL/well keratinocyte serum free media and supplements described earlier.

After verification of fibroblast phenotype by light microscopy, cells were treated with free CTGF-siRNA, CTGF-siRNA in SSLNP, or CTGF-siRNA in SSLNP-GalN at siRNA concentrations of 50, 100 and 200 nM. 50 nM CTGF-siRNA in LF was used as positive control, while 200 nM scrambled siRNA in SSLNP-GalN was used as a negative control.

Treated cells were then washed and re-incubated with fresh media for 48 h to allow the downregulation of CTGF and degradation of collagenous matrix. At the end of incubation period, cells were washed three times with (37 °C) PBS with Ca^2+^ and Mg^2+^ and fixed with ice-cold ethanol for 10 min, then dyed with Sirius red/fast green (Chondrex, Inc., Redmond, WA, USA) according to the supplier’s protocol. Data were expressed as percentage of collagen normalized to non-collagenous proteins, determined as described above for HSC cells.

### 3.8. In Vivo Biodistribution and Pharmacokinetic Studies

Biodistribution studies were performed on healthy 6 weeks old Balb/c male mice. Mice were randomized into four groups of 4 animals/group and treated with one of the following formulations: free Cy5 in 5% dextrose (D5W) (60 µg/animal corresponding to 76 nmol/kg), free Cy5-labeled siRNA, Cy5-labeled siRNA in SSLNP or Cy5-labeled siRNA in SSLNP-GalN with the later three formulations administered at 1mg siRNA/animal corresponding to 76 nmol/kg dose. Formulations were injected via tail vein at 0.1 mL and mice were anesthetized with intraperitoneal (IP) injection using ketamine/xylazine (90 mg/kg/3 mg/kg) then sacrificed by exsanguination at predetermined time points of 15 min, 1 h, 3 h, 6 h, 9 h and 24 h. Organs (heart, spleen, lungs, kidneys, and liver) as well as blood and urine were collected from each animal and photographed using Xenogen (Caliper Life Sciences, MA, USA) IVIS Spectrum 100 imaging system at excitation and emission wavelengths of 640 nm and 680 nm respectively. Fluorescence signals were quantified using Living Image 4.0 acquisition and analysis software, by Caliper Life Sceinces, MA, USA. Blood, collected by cardiac puncture into EDTA coated BD™ microtainer tubes, was centrifuged at 3000 rpm for 10 min to separate plasma. Cy5-siRNA concentration in plasma was quantified using a 96-well plate reader BioTek Synergy 4 (Winooski, VT, USA) at excitation and emission wavelengths of 640 nm and 680 nm respectively. siRNA concentration at tested time points was used to plot plasma concentration *vs.* time curve and calculate PK parameters. Pharmacokinetic parameters were calculated according to two-compartmental modeling using Pharsight Phoenix WinNonlin 6.3 PK/PD modeling and simulation software, by Certara, NJ, USA.

### 3.9. Data and Statistical Analysis

All results are expressed as the mean ± standard deviation (SD) of at least three experiments. For statistical analysis, student’s t-test or one-way analysis of variance (ANOVA) followed by Fisher least significant difference post-hoc test were used. *p* values less than 0.05 (*p* < 0.05) were considered to be statistically significant.

## 4. Conclusions

In conclusion, we have developed and characterized a novel, lipid based nanocarrier for siRNA delivery, which can be targeted to hepatic and renal tissues. The developed siRNA nanomedicine had the right size (<100 nm), protected siRNA from RNAse enzyme, delivered siRNA into hepatic and renal cells showing dose dependent efficacy with low toxicity.

Furthermore, when GalN conjugated siRNA nanomedicine was injected to mice; it mostly delivered siRNA to tissues, which have the target receptors of interest. These results indicate that this nanomedicine is a promising candidate to be utilized in future for the treatment of hepatic and renal tissue fibrosis, which are conditions with increasing incidence in recent years [48]. Furthermore, due to the safety and relative ease of production of the proposed RNAi therapeutic, transition to clinics in a timely manner should not be a serious concern.

## Supplementary Materials

The following are available online at http://www.mdpi.com/2079-4991/6/1/8/s1.

## References

[1] Gavrilov K., Saltzman W.M. (2012). Therapeutic siRNA: Principles, challenges, and strategies. Yale J. Biol. Med..

[2] Lam J.K., Chow M.Y., Zhang Y., Leung S.W. (2015). siRNA *Versus* miRNA as Therapeutics for Gene Silencing. Mol. Ther. Nucleic Acids.

[3] Ozpolat B., Sood A., Lopez-Berestein G. (2010). Nanomedicine based approaches for the delivery of siRNA in cancer. J. Intern. Med..

[4] Ameyar-Zazoua M., Guasconi V., Ait-Si-Ali S. (2005). siRNA as a route to new cancer therapies. Expert Opin. Biol. Ther..

[5] Lin Q., Chen J., Zhang Z., Zheng G. (2014). Lipid-based nanoparticles in the systemic delivery of siRNA. Nanomedicine.

[6] Whitehead K.A., Sahay G., Li G.Z., Love K.T., Alabi C.A., Ma M., Zurenko C., Querbes W., Langer R.S., Anderson D.G. (2011). Synergistic silencing: Combinations of lipid-like materials for efficacious siRNA delivery. Mol. Ther..

[7] Timko B.P., Whitehead K., Gao W., Kohane D.S., Farokhzad O., Anderson D., Langer R. (2011). Advances in drug delivery. Ann. Rev. Mater. Res..

[8] Shi B., Keough E., Matter A., Leander K., Young S., Carlini E., Sachs A.B., Tao W., Abrams M., Howell B. (2011). Biodistribution of small interfering RNA at the organ and cellular levels after lipid nanoparticle-mediated delivery. J. Histochem. Cytochem..

[9] Whitehead K.A., Langer R., Anderson D.G. (2009). Knocking down barriers: Advances in siRNA delivery. Nat. Rev. Drug Discov..

[10] Zuhorn I.S., Engberts J.B., Hoekstra D. (2007). Gene delivery by cationic lipid vectors: Overcoming cellular barriers. Eur. Biophys. J..

[11] Elbashir S.M., Martinez J., Patkaniowska A., Lendeckel W., Tuschl T. (2001). Functional anatomy of siRNAs for mediating efficient RNAi in Drosophila melanogaster embryo lysate. EMBO J..

[12] McCrudden C.M., McCarthy H.O. (2013). Cancer gene therapy-key biological concepts in the design of multifunctional non-viral delivery systems. Gene Ther. Tools Potential Appl..

[13] Alexis F., Pridgen E., Molnar L.K., Farokhzad O.C. (2008). Factors affecting the clearance and biodistribution of polymeric nanoparticles. Mol. Pharm..

[14] Jackson A.L., Bartz S.R., Schelter J., Kobayashi S.V., Burchard J., Mao M., Li B., Cavet G., Linsley P.S. (2003). Expression profiling reveals off-target gene regulation by RNAi. Nat. Biotechnol..

[15] Gao W., Xiao Z., Radovic-Moreno A., Shi J., Langer R., Farokhzad O.C. (2010). Progress in siRNA delivery using multifunctional nanoparticles. Methods Mol. Biol..

[16] Tomar R.S., Matta H., Chaudhary P.M. (2003). Use of adeno-associated viral vector for delivery of small interfering RNA. Oncogene.

[17] Akinc A., Goldberg M., Qin J., Dorkin J.R., Gamba-Vitalo C., Maier M., Jayaprakash K.N., Jayaraman M., Rajeev K.G., Manoharan M. (2009). Development of lipidoid–siRNA formulations for systemic delivery to the liver. Mol. Ther..

[18] Xue H.Y., Liu S., Wong H.L. (2014). Nanotoxicity: A key obstacle to clinical translation of siRNA-based nanomedicine. Nanomedicine.

[19] Yu B., Hsu S.-H., Zhou C., Wang X., Terp M.C., Wu Y., Teng L., Mao Y., Wang F., Xue W. (2012). Lipid nanoparticles for hepatic delivery of small interfering RNA. Biomaterials.

[20] Pecot C.V., Calin G.A., Coleman R.L., Lopez-Berestein G., Sood A.K. (2011). RNA interference in the clinic: Challenges and future directions. Nat. Rev. Cancer.

[21] Marrink S.J., Lindahl E., Edholm O., Mark A.E. (2001). Simulation of the spontaneous aggregation of phospholipids into bilayers. J. Am. Chem. Soc..

[22] Semple S.C., Akinc A., Chen J., Sandhu A.P., Mui B.L., Cho C.K., Sah D.W., Stebbing D., Crosley E.J., Yaworski E. (2010). Rational design of cationic lipids for siRNA delivery. Nat. Biotechnol..

[23] Schroeder A., Levins C.G., Cortez C., Langer R., Anderson D.G. (2010). Lipid-based nanotherapeutics for siRNA delivery. J. Intern. Med..

[24] Phanish M.K., Winn S., Dockrell M. (2009). Connective tissue growth factor-(CTGF, CCN2)—A marker, mediator and therapeutic target for renal fibrosis. Nephron Exp. Nephrol..

[25] Gressner O.A., Gressner A.M. (2008). Connective tissue growth factor: A fibrogenic master switch in fibrotic liver diseases. Liver Int..

[26] Salazar-Montes A.M., Hernández-Ortega L.D., Lucano-Landeros M.S., Armendariz-Borunda J. (2015). New gene therapy strategies for hepatic fibrosis. World J. Gastroenterol..

[27] Hernandez-Gea V., Friedman S.L. (2011). Pathogenesis of liver fibrosis. Annu. Rev. Pathol. Mech..

[28] Luo G., Lu Y., Song J., Yang L., Shi Y., Li Y. (2008). Inhibition of Connective Tissue Growth Factor by Small Interfering RNA Prevents Renal Fibrosis in Rats Undergoing Chronic Allograft Nephropathy. Transpl. Proc..

[29] George J., Tsutsumi M. (2007). siRNA-mediated knockdown of connective tissue growth factor prevents *N*-nitrosodimethylamine-induced hepatic fibrosis in rats. Gene Ther..

[30] Kong W.H., Park K., Lee M.-Y., Lee H., Sung D.K., Hahn S.K. (2013). Cationic solid lipid nanoparticles derived from apolipoprotein-free LDLs for target specific systemic treatment of liver fibrosis. Biomaterials.

[31] Huang Z., Yi B., Yuan H., Yang G. (2014). Efficient delivery of connective tissue growth factor shRNA using PAMAM nanoparticles. Genet. Mol. Res..

[32] Vukovic L., Khatib F.A., Drake S.P., Madriaga A., Brandenburg K.S., Král P., Onyuksel H. (2011). Structure and dynamics of highly PEG-ylated sterically stabilized micelles in aqueous media. J. Am. Chem. Soc..

[33] Lee R.T., Wang M.-H., Lin W.-J., Lee Y.C. (2011). New and more efficient multivalent glyco-ligands for asialoglycoprotein receptor of mammalian hepatocytes. Bioorgan. Med. Chem..

[34] Seow Y., Tan M., Woo K.T. (2002). Expression of a functional asialoglycoprotein receptor in human renal proximal tubular epithelial cells. Nephron.

[35] Moghimi S.M., Andersen A.J., Hashemi S.H., Lettiero B., Ahmadvand D., Hunter A., Andresen T.L., Hamad I., Szebeni J. (2010). Complement activation cascade triggered by PEG-PL engineered nanomedicines and carbon nanotubes: The challenges ahead. J. Control. Release.

[36] Lim S.B., Banerjee A., Önyüksel H. (2012). Improvement of drug safety by the use of lipid-based nanocarriers. J. Control. Release.

[37] Knop K., Hoogenboom R., Fischer D., Schubert U.S. (2010). Poly (ethylene glycol) in drug delivery: Pros and cons as well as potential alternatives. Angew. Chem. Int. Ed..

[38] Li Y., Huang G., Diakur J., Wiebe L.I. (2008). Targeted delivery of macromolecular drugs: Asialoglycoprotein receptor (ASGPR) expression by selected hepatoma cell lines used in antiviral drug development. Curr. Drug Deliv..

[39] Wu Y.T., Jiaang W.T., Lin K.G., Huang C.M., Chang C.H., Sun Y.L., Fan K.H., Hsu W.C., Wang H.E., Lin S.B. (2004). A new *N*-acetylgalactosamine containing peptide as a targeting vehicle for mammalian hepatocytes via asialoglycoprotein receptor endocytosis. Curr. Drug Deliv..

[40] Bumcrot D., Manoharan M., Koteliansky V., Sah D.W. (2006). RNAi therapeutics: A potential new class of pharmaceutical drugs. Nat. Chem. Biol..

[41] Zhang Y., Satterlee A., Huang L. (2012). *In Vivo* Gene Delivery by Nonviral Vectors: Overcoming Hurdles&quest. Mol. Ther..

[42] Mishra S., Webster P., Davis M.E. (2004). PEGylation significantly affects cellular uptake and intracellular trafficking of non-viral gene delivery particles. Eur. J. Cell Biol..

[43] Huang L., Liu Y. (2011). *In vivo* delivery of RNAi with lipid-based nanoparticles. Annu. Rev. Biomed. Eng..

[44] Tryggvason K., Wartiovaara J. (2005). How does the kidney filter plasma?. Physiology.

[45] Ashok B., Arleth L., Hjelm R.P., Rubinstein I., Önyüksel H. (2004). *In vitro* characterization of PEGylated phospholipid micelles for improved drug solubilization: Effects of PEG chain length and PC incorporation. J. Pharm. Sci..

[46] Dagar A., Kuzmis A., Rubinstein I., Sekosan M., Onyuksel H. (2012). VIP-targeted cytotoxic nanomedicine for breast cancer. Drug Deliv. Transl. Res..

[47] Rhyu D.Y., Yang Y., Ha H., Lee G.T., Song J.S., Uh S.T., Lee H.B. (2005). Role of reactive oxygen species in TGF-β1-induced mitogen-activated protein kinase activation and epithelial-mesenchymal transition in renal tubular epithelial cells. J. Am. Soc. Nephrol..

[48] Patel J., Gupta S., Fauzdar M., Patel N., Chaturvedi S. (2015). Congenital Hepatic Fibrosis Associated with Polycystic Kidney Disease. J. Liver.

