# Supplementary material for: Targeted Sterically Stabilized Phospholipid siRNA Nanomedicine for Hepatic and Renal Fibrosis
Source: Nanomaterials (Basel). 2016 Jan 5;6(1):8. doi: 10.3390/nano6010008 (PMC5302539; doi:10.3390/nano6010008)
Supplement: Supplementary file 1 [file nanomaterials-06-00008-s001.pdf]

Supplementary Material

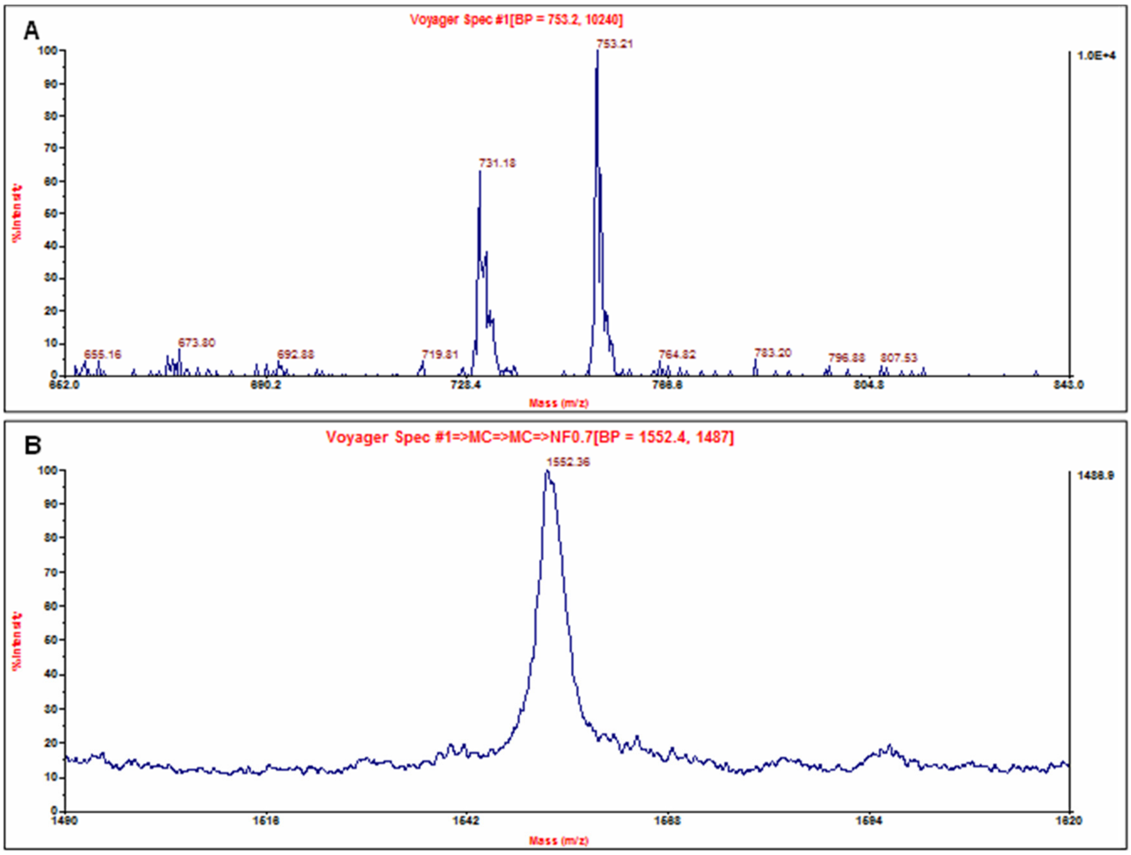

Figure S1. Accumulated mass spectrum (100 shots/ spectra) of (**A**) Ptdthioethanol lipid; and (**B**) cationic lipid-Z, with shift in molecular weight confirming 4R peptide conjugation.

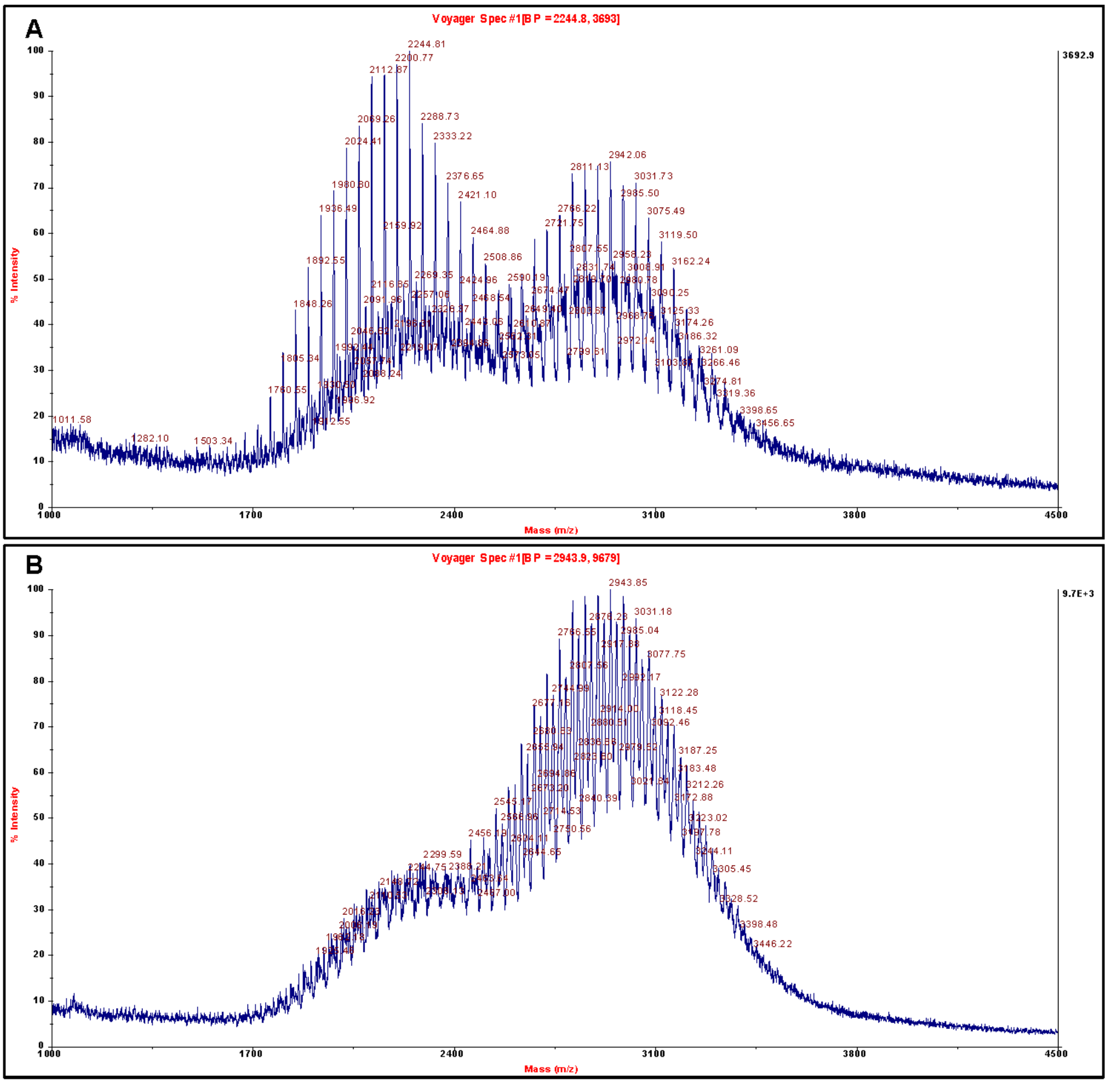

Figure S2. Mass spectrum of (**A**) free DSPE-PEG2000-COOH; and (**B**) conjugated DSPE-PEG2000-Galactosamine showing a shift in molecular weight confirming conjugation.

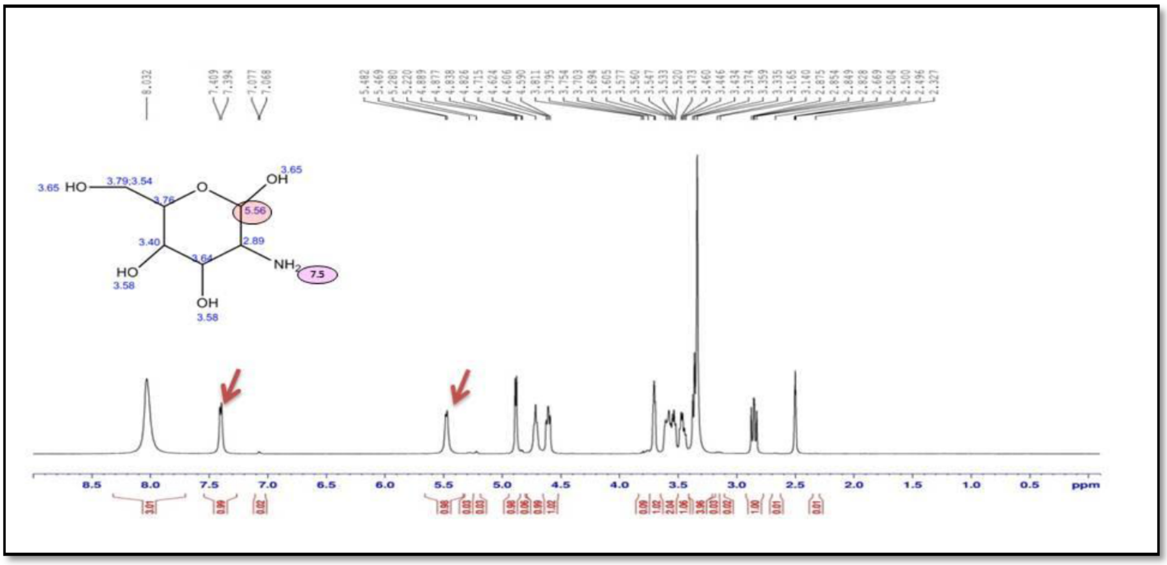

Figure S3. *Cont.*

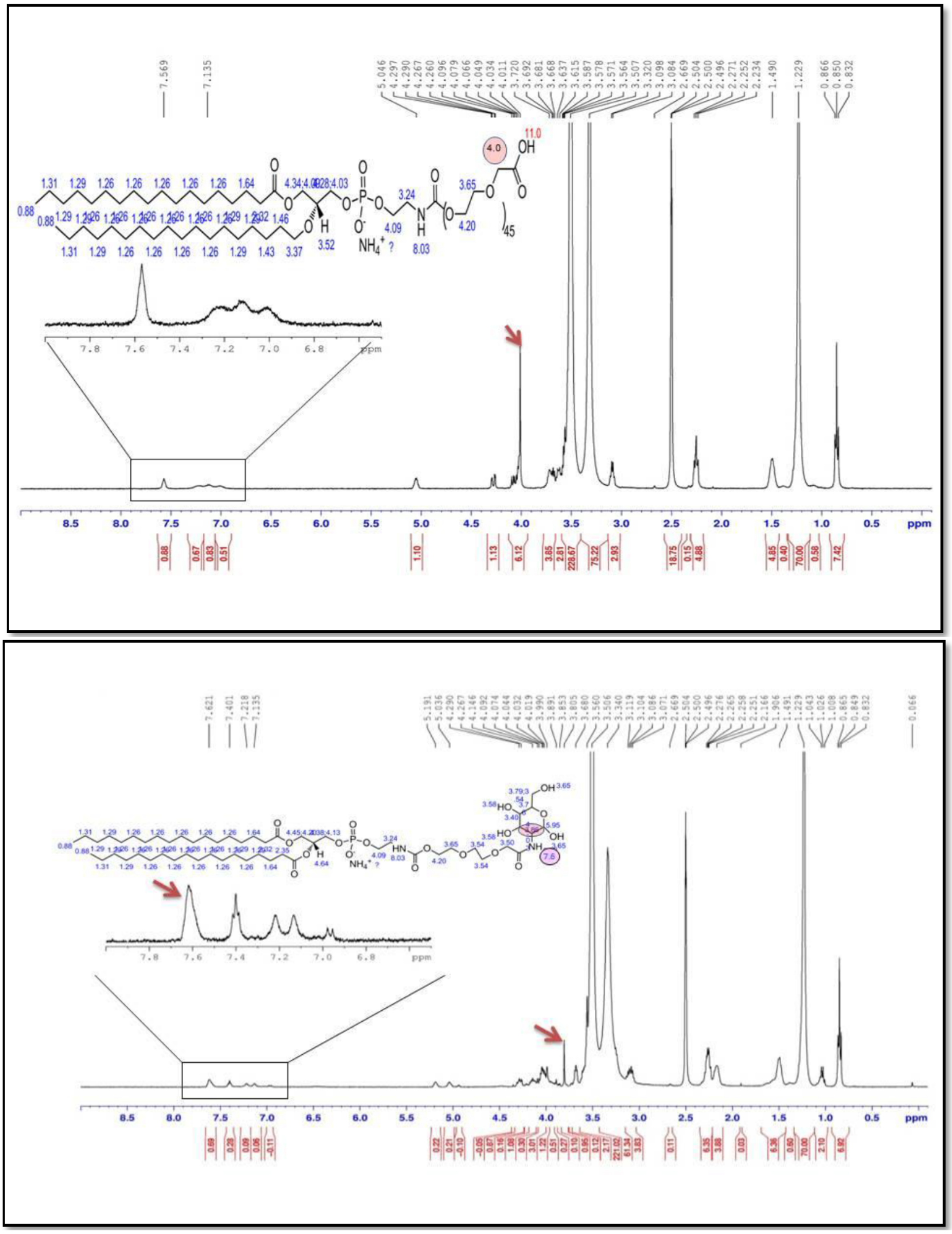

Figure S3. NMR spectrum of D-galactosamine (upper spectrum), DSPE-PEG2000-COOH (middle spectrum) and DSPE-PEG2000-Galactosamine (lower spectrum) with peak shifts indicating successful conjugation. Percent conjugation was calculated from spectra to be ~30%.

© 2016 by the authors; licensee MDPI, Basel, Switzerland. This article is an open access article distributed under the terms and conditions of the Creative Commons by Attribution (CC-BY) license (http://creativecommons.org/licenses/by/4.0/).
